# Teenagers with Obesity at the Gym: Recommendations for Physical Activity, Diet, and Supplementation—A Narrative Review

**DOI:** 10.3390/nu17111798

**Published:** 2025-05-26

**Authors:** Agnieszka Kozioł-Kozakowska, Małgorzata Wójcik, Paulina Mazur-Kurach, Dorota Drożdż, Anna Brzęk

**Affiliations:** 1 Department of Pediatrics, Gastroenterology and Nutrition, Institute of Pediatrics, Faculty of Medicine, Medical College, Jagiellonian University, Wielicka 265 St., 30-663 Cracow, Poland; 2 Department of Pediatric and Adolescent Endocrinology, Chair of Pediatrics, Institute of Pediatrics, Faculty of Medicine, Medical College, Jagiellonian University, Wielicka 265 St., 30-663 Cracow, Poland; malgorzata.wojcik@uj.edu.pl; 3 Department of Sports Medicine and Human Nutrition, Institute of Biomedical Sciences, University of Physical Culture, al. Jana Pawla II 78, 31-571 Cracow, Poland; paulina.mazur@awf.krakow.pl; 4 Department of Pediatric Nephrology and Hypertension, Chair of Pediatrics, Institute of Pediatrics, Medical College, Jagiellonian University, Wielicka 265 St., 30-663 Cracow, Poland; dorota.drozdz@uj.edu.pl; 5 Department of Physiotherapy, Faculty of Health Sciences in Katowice, Medical University of Silesia, Medyków 12 St., 40-756 Katowice, Poland; abrzek@sum.edu.pl

**Keywords:** child nutrition, obesity, diet supplementation, physical activity

## Abstract

Child and adolescent obesity represents a growing public health crisis, with profound implications for physical and mental well-being. Despite the well-established benefits of physical activity, over 80% of adolescents fail to meet the World Health Organization’s (WHO) recommendations for daily exercise. This manuscript explores gym-based strength training as a tailored intervention for obese youth who often struggle with aerobic activities. This paper critically examines medical eligibility, safety protocols, and injury risks while also detailing personalized training regimens that prioritize neuromuscular adaptation, functional strength, and long-term engagement. Additionally, it integrates evidence-based dietary strategies and supplementation practices appropriate for this age group, highlighting the role of the Mediterranean diet, DASH, and the cautious use of supplementation, particularly probiotics and protein, while discouraging performance-enhancing substances in youth. Emphasis is placed on interdisciplinary approaches, combining exercise, nutrition, and medical oversight to support holistic treatment. This study identifies gaps in the current literature and provides practical recommendations for designing safe, effective, and sustainable gym-based interventions for children with obesity, encouraging further research to validate these protocols in clinical settings. A comprehensive search was conducted across multiple databases, including PubMed/MEDLINE, the Cochrane Library, Science Direct, and EBSCO, focusing on English-language meta-analyses, systematic reviews, randomized clinical trials, and observational studies. The websites of prominent scientific organizations such as WHO, APP, and AIS were also reviewed. The selection of articles was a collaborative effort among all authors.

## 1. Introduction

Obesity in children and adolescents is a growing public health concern, associated with numerous physical and psychological health issues. According to the World Health Organization, over 340 million children and adolescents aged 5–19 were classified as overweight or obese—a figure that has more than quadrupled since 1975. The prevalence continues to rise across both high-income and low- to middle-income countries, signaling a widespread public health challenge. This alarming trend underscores the urgent need for innovative, sustainable lifestyle interventions that are specifically tailored to youth populations [1,2]. Developing the habit of regular physical activity is one of the pillars of lifestyle intervention in this age group. Physical exercise is a means used in physioprophylaxis for many diseases. During the developmental age, physical activity (PA) is essential for proper psychomotor development. Although this is not a matter of debate today, the recommendations of the World Health Organization (WHO) in this regard are not being met worldwide. For adolescents, this means at least 60 min of moderate to vigorous physical activity per day [3]. Scientific studies indicate a link between low levels of physical activity and poor physical fitness, which is a strong, independent predictor of health status and functional fitness in adulthood [4,5].

The WHO recommendations are also driven by alarming statistics on obesity among children and adolescents worldwide [6] as a means of preventing serious cardiovascular complications of obesity, such as an increased risk of hypertension [7], metabolic syndrome, dyslipidemia [8], and insulin resistance [9]. Additionally, obesity negatively impacts mental health, manifesting as lower self-esteem and a higher risk of anxiety [10,11,12]. It is also known that overweight and obese children tend to spend their free time in a sedentary manner more often [11,13,14]. Despite the well-documented benefits of physical activity, research shows that over 80% of adolescents aged 11–17 years are insufficiently physically active, and overweight and obese children tend to spend more time in sedentary activities [15]. The situation is even more critical for pediatric patients who are overweight and obese. Any form of exercise and even the smallest reduction in a sedentary lifestyle is crucial in the treatment of obesity in children and adolescents. However, this group often has special requirements, showing reluctance to engage in physical activity and a higher susceptibility to injuries compared to their peers. Strength training may be particularly beneficial for obese youths who may not tolerate prolonged periods of aerobic exercise [16]. It can serve as a first step towards changing attitudes towards physical conditioning, fitness, and lifestyle modification. Effective strength training programs for adolescents should include a systematic approach, periodization, adequate recovery time, the gradual modification of exercises and their intensity, and the maintenance of achieved effects during the stabilization phase [17]. A well-balanced diet rich in natural nutrients is crucial for physically active children with obesity. Any changes to the diet should be discussed with a doctor or dietitian to suit the individual needs of the child. Proper nutrition supports physical fitness, healthy weight reduction, and prevents nutritional deficiencies.

Despite a growing body of literature on pediatric obesity, most existing studies focus broadly on clinical interventions, school-based programs, or community-level strategies rather than gym-based settings. While these approaches have demonstrated some success in addressing obesity, they often lack the specificity and contextual relevance required for adolescents who choose or are prescribed structured exercise in gym environments.

Moreover, the majority of gym-based physical activity studies either focus on adult populations or treat youth as a homogeneous group, failing to differentiate between overweight/obese and healthy-weight individuals. As a result, they overlook the unique challenges that obese teens face, such as greater susceptibility to injury. There is also a lack of tailored guidance on how to progress exercise regimens safely and effectively for this demographic, especially in the absence of clinical supervision. In the area of dietary intervention, existing recommendations are often generalized, lacking integration with exercise routines typically performed in gym settings. They rarely consider the socio-emotional context of obese teens, who may struggle with body image issues or disordered eating patterns. Similarly, evidence on supplementation is inconclusive for this age group, with most studies conducted on adults or athletes, rendering it difficult to translate findings into safe and effective protocols for adolescents.

Another critical gap lies in interdisciplinary approaches. Few studies offer coordinated guidance that synthesizes physical activity, diet, and supplementation specifically for obese teenagers engaging in gym-based programs. This siloed research landscape contributes to fragmented and often conflicting recommendations, making it harder for practitioners, trainers, and families to support these youths effectively.

Through this manuscript, we want to respond to the following key questions: What specific types and structures of gym-based physical activity programs are most effective and sustainable for obese teenagers?

How can dietary strategies be tailored to support adolescents engaged in gym-based fitness routines, considering their unique metabolic and psychosocial needs?

Are there safe, evidence-based supplementation protocols that can complement physical training in obese teens, and what are the potential risks?

Addressing these questions is essential for developing comprehensive, evidence-informed recommendations that align with the realities faced by obese teenagers in gym environments.

## 2. Materials and Methods

The research involved a comprehensive search across multiple databases, including PubMed/MEDLINE, the Cochrane Library, Science Direct, and EBSCO. The focus was on English-language meta-analyses, systematic reviews, randomized clinical trials, and observational studies from around the globe. Additionally, the websites of prominent scientific organizations such as the World Health Organization (WHO), American Academy of Pediatrics (APP), and Australian Institute of Sport (AIS) were reviewed. The selection of articles was a collaborative effort among all authors.

## 3. Medical Aspects of Gym Training in Adolescence

The issue of physical activity in children with obesity is challenging. Sometimes, the only form of exercise that overweight and obese teenagers are willing to engage in and find interesting is strength training. This type of activity may be particularly beneficial for youths with obesity, as they often struggle to tolerate prolonged periods of aerobic exercise [18]. Strength training, as part of a structured exercise program, can contribute to weight management and improve body composition [19]. It may also enhance muscular strength and endurance, enabling participation in other forms of physical activity. In the long term, it can help reduce body fat and increase lean muscle mass [20]. Positive effects on lipid profiles and blood pressure have also been observed [21,22].

Although professional and safe training yields significant benefits and poses no threat to health, allowing an obese child to exercise in a gym still raises concerns among parents and pediatricians. Experts emphasize that every child with obesity should undergo a medical examination before beginning strength training. This initial assessment is necessary to identify any contraindications and evaluate the potential risks. Strength training is generally not recommended for children and adolescents with uncontrolled hypertension, seizure disorders, or a history of anthracycline-based chemotherapy for childhood cancer without additional medical evaluation and/or treatment [18]. Strength training is contraindicated in patients with cardiomyopathy (particularly hypertrophic), as it may worsen ventricular hypertrophy or lead to hemodynamic decompensation due to an acute increase in pulmonary hypertension. Adolescents with moderate to severe pulmonary hypertension should avoid strenuous weight training, as they are at risk of acute cardiovascular decompensation [18].

In addition to identifying potential contraindications, the initial medical examination provides an opportunity to assess the child’s overall health, offer guidance, set realistic expectations, dispel common myths, and address general concerns about strength training in children. It also allows healthcare providers to discuss health behaviors and educate both the child and parents [23]. There is no minimum age requirement for participation in strength training; however, children must demonstrate sufficient maturity to follow instructions, typically around the age of seven or eight. A fundamental requirement for qualifying an obese child for strength training is their understanding of the principles, goals, and importance of this type of exercise. The decision should be based on the child’s physical, cognitive, and social maturity [24].

Children and adolescents should only begin strength training if they perceive it as beneficial and should continue only if they find it enjoyable and satisfying. One of the key factors influencing the medical evaluation is the child’s and parents’ motivation and expectations. It is crucial for them to understand that the primary objectives of strength training are to enhance muscular strength and endurance rather than to increase muscle size [25].

In both prepubertal children and adolescents, strength training effectively increases muscle strength. However, it is important to note that significant muscle hypertrophy is not a typical response to strength training in prepubertal children [26]. Strength training improves the coordinated recruitment of motor units, increases the number of motor units activated, and enhances the firing rate and pattern of motor neurons [27]. These neuromuscular adaptations contribute to strength gains and may help prevent injuries in other sports and daily activities [28].

A common concern regarding strength training in children is the potential risk of epiphyseal (growth plate) damage. Older studies from the 1970s and 1980s suggested that strength training could negatively affect growth due to growth plate injuries [29]. However, more recent research has shown that with proper supervision and adherence to safety guidelines, the risk of such injuries is minimal.

Data from injury surveillance studies conducted between 2003–2005 and 1990–2007 indicate that most weight training-related injuries in children involve the hands and feet and result from accidents, such as dropping weights. These injuries are largely preventable with adequate supervision and adherence to safety protocols [30]. Proper coaching and responsible supervision are crucial in minimizing the risk of strength training-related injuries in youth. A well-structured and supervised strength training program is no more dangerous (and often safer) than many other recreational activities or sports. When performed with appropriate frequency, intensity, and technique, strength training does not place excessive stress on the growth plates and does not impair growth. On the contrary, it may positively impact bone development during childhood and adolescence [31].

Another concern is the potential risk of repetitive soft tissue injuries and lower back injuries in children participating in strength training. These injuries often result from using unsafe training equipment, poor lifting techniques, or lifting weights that are too heavy [30]. To minimize these risks, children should always perform exercises under adult supervision. Coaches and trainers should have the necessary knowledge and experience to develop and monitor a safe and effective strength training program. In a recently published study, the risk factors of injury during exercise were described. A significant statistical association was found between injury prevalence and age, gender, educational level, period of training, and presence of a trainer. However, no association was detected between injury prevalence and body mass index (BMI), place of residence, or source of advice [32].

It is also important to recognize that obese children often have various cardiovascular risk factors, which may predispose them to long-term health complications. A prospective cohort study found that childhood risk factors—including a high body mass index (BMI), elevated systolic blood pressure, high total cholesterol and triglyceride levels, and smoking—were associated with cardiovascular events in adulthood [33].

When recommending physical activity to adolescents with obesity, it should be borne in mind that every third person in this group has hypertension. As a group at risk of developing hypertension, blood pressure (BP) measurements should be performed at every medical visit. Office BP measurement is recommended for screening, diagnosis, and management of high BP in children and adolescents, and validated devices for children should be used. In obese children, care should be taken to select the appropriate cuff size. If elevated blood pressure values are detected in office or home measurements, an obese teenager should be referred to a specialist. Moreover, 24 h ambulatory blood pressure monitoring (ABPM) is recommended to confirm hypertension and avoid treatment for white-coat hypertension. ABPM also allows the detection of masked hypertension and the assessment of the circadian BP rhythm [34].

Periodic BP monitoring will allow the detection of elevated BP values and, in the case of lifestyle modification with weight loss, the assessment of the effectiveness of non-pharmacological treatment. In young people with stage 2 hypertension, organ damage, or a lack of success with lifestyle modification, antihypertensive treatment should be initiated [35].

Physical activity is one of the main pillars of non-pharmacological intervention in adolescents with hypertension and the basis of obesity prevention and treatment programs.

The contemporary literature emphasizes that physical exercise, including training, is recommended for children and adolescents with obesity in order to reduce body fat and improve physical fitness, which, in turn, decreases the risk of obesity-related comorbidities, including type 2 diabetes and cardiovascular diseases [36]. Previous studies by Davis et al. [37] demonstrated the effectiveness of aerobic exercise interventions in reducing body fat, whereas studies by Damaso et al., analyzing resistance or combined training, did not show significant reductions in either body fat or fat-free mass [38]. From a practical standpoint, it is important that a specialist clearly defines training parameters such as duration and frequency. In this regard, several studies conducted by researchers from Canada [39,40], Australia [41], and Norway [42] indicate the effectiveness of multi-week interventions performed 3–5 times per week for 30 to 60 min per session, at moderate to vigorous intensity. These interventions most commonly involve aerobic, resistance, or combined aerobic and resistance exercises. A meta-analysis of 27 studies conducted by Lee in 2020 showed that aerobic training has moderate effectiveness in treating obesity, while resistance training did not demonstrate such remarkable results [43]. In the authors’ opinion, combining resistance and aerobic training in gym-based programs is a beneficial approach.

## 4. Recommendations for Physical Activity

**Figure 1:**
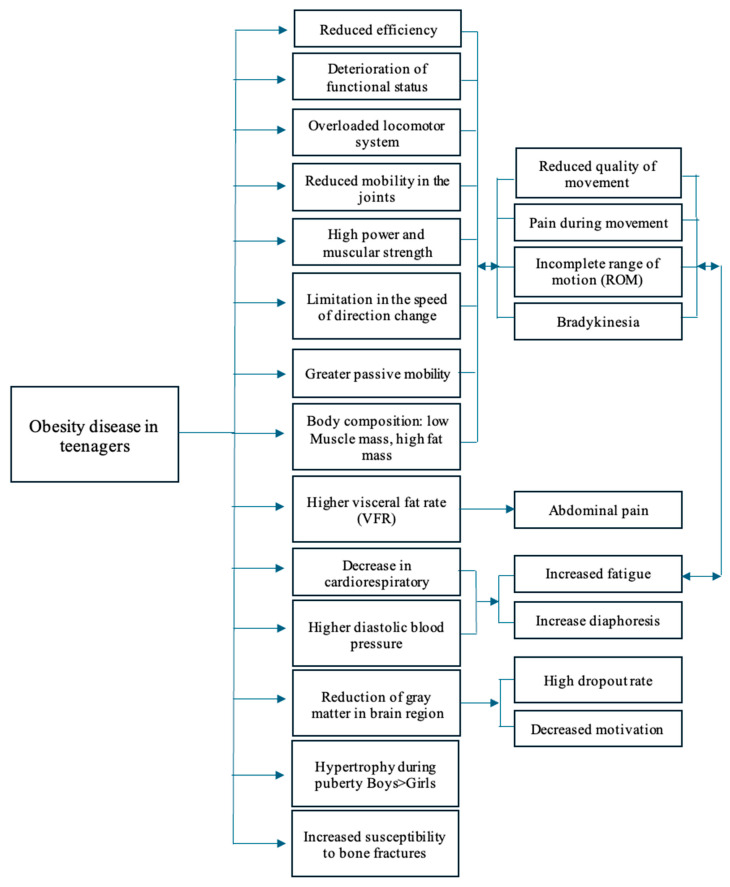
Interactions between the effects of the disease and probable symptoms during physical exertion [44,45,46,47,48,49,50,51,52].

While the link between obesity and physical fitness is well established, there is still limited knowledge regarding the types, frequency, and intensity of physical exercise suitable for adolescents with obesity. The first step is to identify weak points that may affect the effectiveness of recommended gym exercises. Understanding these factors will enable the development of a personalized training program tailored to the specific needs of each adolescent, complementing their treatment process, including physiotherapy (Figure 1).

Over the past decade, there has been a noticeable increase in the implementation of strength training programs in youth sports, highlighting potential health benefits, improved fitness, and a reduced risk of cardiovascular diseases [53], while also emphasizing its impact on mental well-being [6,54]. Scientific evidence and recommendations from scientific organizations, such as the American Academy of Pediatrics Council on Sports Medicine and Fitness [55], indicate that strength training is both effective and safe for children and adolescents.

Strength training is particularly beneficial for treating overweight or obese children and adolescents, as it improves body composition by reducing fat mass, especially in central areas, and is also associated with increased insulin sensitivity in overweight teenagers [56]. Given that obesity itself is a risk factor for cardiovascular diseases, combining resistance training with aerobic exercises has significant cardioprotective benefits, similar to its role in cardiac rehabilitation [57].

However, the overriding condition is its proper selection and supervision [58]. What does this mean in clinical practice? The following factors should be included: (1) a systematic approach; (2) periodization; (3) time for recovery; (4) the gradual modification of exercises and their intensity, considering the principles of progressive overload and individualization; (5) and the maintenance of achieved effects during the stabilization phase after reaching the so-called plateau phenomenon [59].

To achieve the above, training should follow the traditional structure of an exercise session, consisting of a warm-up, main part, and cool-down. Literature data were collected to provide appropriate recommendations for each phase, serving as guidelines for professionals working with individuals affected by obesity. The warm-up should be based on aerobic exercises, whose beneficial effects have been well documented, while the cool-down phase should focus on calming the body and normalizing physiological parameters such as the heart rate (HR), systolic blood pressure (SBP), diastolic blood pressure (DBP), and respiration [41,60]. The main part of a combined training session should be grounded in scientific research that defines the recommended training duration, intensity, number of repetitions [40,41], the targeted muscle areas [61], and the equipment and tools used during the session [62,63].

The effectiveness of training becomes visible after approximately eight weeks of consistent effort. However, it is important to remember that discontinuing training for 8–12 weeks leads to a return to baseline values [64]. Numerous scientific studies over the past 10 years indicate a long duration of intervention, extending up to 32 weeks in studies conducted by Spanish researchers Serra-Payá et al. [65] and Williams et al. [63]. However, the most common duration is 12 weeks [42,66]. This is particularly relevant in cases where a temporary training interruption is necessary due to illness or unavailability. The timing of returning to training should be determined in consultation with a physician and a physiotherapist to ensure that the break is not too long and that resuming strength exercises does not disrupt the healing process.

Since gym-based training involves various types of exercises depending on the training plan and set goals, and despite recommendations that children and adolescents should complement strength training with motor skills such as jumping and running, these activities should not be implemented in children with obesity, particularly at the initial stage of training. This type of physical activity may contribute to damage to the growth cartilage [28]. The reason for this lies in excessive strain on peripheral joints (knee and ankle) and the spinal joints, as well as a higher susceptibility to fractures of long bones [67,68,69].

In the professional community, there is a widespread belief that any physical activity is better than physical inactivity. However, it is important to emphasize that obesity is a disease with numerous consequences for the musculoskeletal system, including overload of peripheral joints and the spine. Additional uncontrolled loads or weight-bearing movements may lead to dysfunctions, injuries, and limitations in physical activity, which is undesirable in the context of obesity.

Considering research findings that indicate more complex injury patterns in adolescents with obesity [70] and the increased risk of complications in orthopedic injuries and delayed bone healing reported by Fornari et al. [71], the implementation of supervised training—at least during the learning phase of specific exercises and positions—appears justified. Such an approach serves as physioprophylaxis against injuries. Research by Schranz et al. [72] on resistance training has shown that it is not harmful during growth, provided that children and adolescents who are overweight and obese are properly supervised by qualified personnel. However, there is a lack of recently published studies on this topic [73].

It is also worth noting certain inconsistencies—for example, the study by Tan et al. [74] bases its training program for 9–10-year-old children who are overweight and obese on jumping and running exercises but fails to specify the surface used. Therefore, the authors of this paper have refined the details concerning proper body positioning, the quality of the exercises performed, and their intensity, while maintaining existing, evidence-based recommendations for physical activity.

To minimize undesirable symptoms occurring during physical exertion, training sessions should follow the scheme presented in Table 1. Naturally, the methods, forms, and means of individual training components should be tailored to each person in collaboration with a qualified personal trainer but only after consulting with the attending physician and physiotherapist. The absence of contraindications is a prerequisite for initiating this type of physical activity. Table 1 presents recommendations for strength training in adolescents, along with modifications for patients with obesity, considering certain limitations described in Figure 1. The authors of this study refer to the work of Faigenbaum et al. [30], which, although lacking specific details on training planning for obese adolescents, can serve as a guideline for designing individualized strength training programs that include essential information for developmental age.

Among the general contraindications for gym training, the following should be noted: (1) Avoid performing exercises independently, such as on machines at home or in the gym—improper posture, incorrect number of repetitions, and inappropriate intensity without considering contraindications can lead to injuries or ineffective training. (2) Avoid anaerobic exercises and plyometric training (SSC—Stretch-Shortening Cycle), as these are recommended at a later stage. The latter is not contraindicated in itself but quickly induces fatigue, which may discourage individuals from this type of training. (3) Avoid exercises that cause muscle imbalances. There is also no scientific data on muscle power development in teenagers with obesity. However, clinical experience suggests that professional supervision and control by qualified gym staff are essential during training.

## 5. Diet and Supplementation

In overweight children undertaking physical activity, a varied, well-balanced diet should provide all the nutrients necessary to maintain and improve health and the ability to exercise and recover. A diet used in obesity treatment can be characterized as a hypocaloric diet providing an age-appropriate amount of macronutrients (proteins, fats, carbohydrates) that is high in dietary fiber, limited in certain types of carbohydrates (added sugars) and fats (saturated and trans fats), and has no highly processed foods. It is worth noting that some popular dietary models, such as vegetarian, Mediterranean, or DASH diets, can also be successfully applied to pediatric patients, provided they meet the above conditions. The advantages of these diets include ensuring an adequate intake of fresh fruits and vegetables (with an emphasis on the latter), thereby covering the necessary dietary fiber requirements, emphasizing fresh, unprocessed foods, which help avoid pro-inflammatory components (such as trans fatty acids) and eliminate calorie-dense, nutrient-poor foods from a child’s diet. Maintaining a low glycemic index and glycemic load due to low simple sugar intake (limiting added sugars, eliminating high-fructose corn syrup) and a high intake of complex carbohydrates (whole-grain products, legumes) and fiber is essential. Additionally, consuming an adequate amount of protein from a diverse range of sources (for instance, Mediterranean diets; legumes and soy products in vegetarian diets; and fish, legumes, poultry, and dairy in DASH (Dietary Approaches to Stop Hypertension)), ensuring a balanced intake of saturated and polyunsaturated fatty acids (omega-3/omega-6) and delivering high amounts of vitamins and antioxidants [80] are also important. The recommendation of specific dietary models such as the Mediterranean, DASH, or vegetarian diets for obese youth is supported not only by their ability to promote healthy weight management but also by their effects on key metabolic and inflammatory pathways that contribute to obesity-related complications. The Mediterranean diet, rich in monounsaturated fats (primarily from olive oil), polyunsaturated fats (from nuts and fish), fiber, and antioxidants from fruits and vegetables, has demonstrated benefits in improving insulin sensitivity and reducing chronic inflammation. Bioactive compounds such as polyphenols (e.g., resveratrol and oleuropein) have been shown to suppress the expression of pro-inflammatory cytokines, like IL-6 and TNF-α, while enhancing the production of anti-inflammatory mediators, such as IL-10 [81,82]. The DASH diet, originally designed to manage hypertension, emphasizes a high intake of fruits, vegetables, whole grains, and low-fat dairy products. Due to its high content of potassium, magnesium, and dietary fiber, it supports vascular health, lowers blood pressure, and improves glucose metabolism by reducing postprandial insulin spikes. Moreover, its antioxidant-rich composition helps mitigate oxidative stress, a factor that contributes to adipose tissue dysfunction and insulin resistance [83]. A vegetarian diet, which eliminates meat and is based primarily on whole plant foods, also exhibits anti-inflammatory and metabolic benefits. The reduction in saturated fat and endotoxins from animal products leads to decreased activation of toll-like receptor 4 (TLR4), thereby lowering both local and systemic inflammation. Additionally, high fiber intake promotes the growth of beneficial gut microbiota, resulting in increased production of short-chain fatty acids such as butyrate, which have been shown to enhance insulin sensitivity and exert anti-inflammatory effects. Plant-based diets can also influence hormonal regulation by improving leptin and adiponectin levels, enhancing satiety, and promoting lipid oxidation [84,85]. In summary, these dietary models recommended for obese youth exert multi-faceted effects that go beyond simple calorie restriction. They act at the molecular level to improve insulin signaling, reduce chronic low-grade inflammation, and support a healthy gut microbiome, all of which contribute to a reduced long-term risk of cardiometabolic diseases.

In specific, justified cases, particularly during periods of intense physical exertion or when there are nutrient deficiencies affecting normal developmental processes, supplementation may be considered. However, it is essential to adhere to the principle that the foundation of rational nutrition should be food with high nutritional value. Overweight or obese children often exhibit nutritional imbalances and deficiencies in certain nutrients due to diets high in processed foods, which lack essential micro- and macronutrients [86]. Effective weight reduction in children and adolescents necessitates maintaining a negative energy balance while considering the nutritional requirements associated with their developmental processes. The inclusion of caloric supplements (e.g., protein supplements, isotonic drinks) can impede weight loss if not properly balanced within the diet [87].

According to the recommendations of the American Academy of Pediatrics (AAP) and the Australian Institute of Sport (AIS), dietary supplements aimed at enhancing athletic performance are generally not advised for physically active children, particularly those under the age of 15, except in cases of identified deficiencies such as vitamin D, iron, and omega-3 fatty acids [18,88].

Supplementation in overweight and obese children who are beginning physical activity requires thorough analysis, considering both potential benefits and numerous challenges related to the safety, efficacy, and regulation of supplements. Creatine is one ergogenic aid considered for physically active children and adolescents [89]. Creatine monohydrate, the most extensively studied form, increases phosphocreatine levels in muscles, potentially enhancing anaerobic performance [89,90,91]. Despite its relative safety in adults, there is no evidence supporting its beneficial effects in overweight and obese children. Some studies report adverse effects of creatine use, including gastrointestinal issues, significant weight gain, and kidney disorders. Creatine can promote muscle strength development in children, but its use remains controversial. Although the physiological basis for the ergogenic benefits of creatine supplementation in adolescents is similar to those observed in adults, the lack of randomized controlled trials and reliable clinical data supporting the safety of these protocols in the adolescent population means that its use should only be under the close supervision of a specialist [86,89,92]. The unsubstantiated ingestion of supplements by adolescents with the hope of improving exercise capacity may perpetuate the belief that better performance is due to supplements. Accordingly, the AAP strongly discourages the use of such substances in adolescent children [93]. Amino acids and proteins represent another category of supplements for physically active children with obesity. Research suggests that consuming amino acids, proteins, and carbohydrates before training can enhance the anabolic response, potentially improving the exercise capacity of children with obesity [94,95]. However, no standardized recommendations have yet emerged regarding the recommended amount of protein intake by obese children and adolescents participating in sports. The most commonly quoted value is 1–1.2 g of protein for each kilogram of body weight. Alternatively, it can be assumed that protein is to realize 15–20% of the energy content of the diet. However, this depends on the sport being practiced. In strength and speed/exercise disciplines, a higher supply is recommended, at up to 2 g per kilogram of body weight, while in endurance disciplines, a lower supply of 1.3 g per kilogram of body weight is recommended [96,97]. Whey protein is particularly valuable for young bodies, as it contains high levels of essential and branched-chain amino acids (BCAAs). Whey protein is highly beneficial for building muscle mass in physically active children and provides antibodies that boost immunity, which can be weakened during intense exercise. However, excessive protein intake, including through supplements, can increase the burden on the kidneys and liver, a concern for children with obesity [96]. Therefore, protein and amino acid supplementation should be tailored to the individual energy and health needs of children to avoid excessive calorie intake and potential metabolic complications [94,98]. It is important to note that with a well-balanced diet and the appropriate selection of natural protein sources, there is generally no need to supplement the diet of physically active children with protein supplements [99].

The diet of physically active children necessitates an adequate intake of fluids, particularly during exercise, when there is significant loss of sweat, along with water and electrolytes. For exercise lasting less than 60 min, water consumption is recommended. For longer workouts, specialized sports drinks that provide water and electrolytes or water and energy are advised. Insufficient fluid intake during increased physical activity can lead to impaired physical performance, manifesting as weakened muscle metabolism, difficulties in thermoregulation, and cardiovascular dysfunction [100,101,102,103]. During exercise lasting < 60 min, mineral water, such as medium or highly mineralized water, is recommended, and during prolonged exercise, drinks designed for athletes (isotonic drinks), which further stimulate drinking due to their sodium content, which acts on osmoreceptors, are recommended. Isotonic drinks for athletes (270–330 mOsm/L) provide potassium and carbohydrates (6–8%; usually glucose) in addition to water and sodium (460–1150 mg/L). Their use should be limited only to hydration during prolonged exercise and in the early post-exercise period, possibly also before prolonged exercise [104,105,106]. However, excessive consumption of sports drinks can increase sugar and energy intake, potentially leading to weight gain and tooth enamel erosion [87].

In the context of weight loss supplements such as green tea and caffeine, the potential benefits may be outweighed by the risk of side effects. Green tea extract, containing EGCG (Epigallocatechin gallate), accelerates weight reduction in adults, but there is insufficient evidence regarding its effectiveness in obese children [107]. Caffeine is a stimulant that, by increasing the release of adrenaline and serotonin, has a stimulating effect on the central nervous system. Some studies on caffeine also confirm its beneficial effects on metabolism and accelerated fat reduction. The increase in adrenaline due to caffeine enhances stamina and concentration. The American Academy of Pediatrics (AAP) recommends that children under the age of 12 should avoid caffeine altogether, while adolescents aged 12–18 should limit their intake to a maximum of 100 mg per day. It is worth noting that caffeine is not only found in coffee but also in certain foods and supplements used by young athletes. Energy drinks, which are popular among young people, contain large amounts of sugar in addition to caffeine and can cause heart rhythm disturbances, shortness of breath, and even impaired consciousness [86,92,107,108].

Regular physical activity in overweight and obese children not only promotes weight reduction but also influences the composition of the gut microbiota, enhancing its diversity and function [109]. In this context, probiotic supplementation may represent a promising therapeutic strategy to support gut microbiota balance and metabolic modulation in children and adolescents [110].

Probiotics, defined as live microorganisms with health benefits, can counter obesity-related dysbiosis by improving intestinal barrier function and influencing satiety in children and adolescents [111,112]. The use of probiotics, particularly in combination with physical activity, can support the body’s energy homeostasis and regulate the secretion of enteroendocrine hormones such as GLP-1 and PYY, which are involved in appetite control and glucose and lipid metabolism [113,114]. Furthermore, appropriately selected probiotic strains can positively influence digestive processes and nutrient absorption, which is crucial for weight reduction [115].

The inclusion of probiotic supplementation in children with obesity can be an inexpensive and effective method of supporting treatment, especially when combined with regular physical activity. *Lactobacillus rhamnosus GG*, used alone or with other strains of the genera Bifidobacteria and Lactobacillus, has been shown to be effective in reducing the BMI and waist circumference [116]. Other beneficial probiotic strains for reducing the BMI include *Streptococcus thermophilus*, *Bifidobacterium breve*, *Bifidobacterium infantis*, *Bifidobacterium longum*, *Lactobacillus acidophilus*, *Lactobacillus plantarum*, *Lactobacillus paracasei* and *Lactobacillus delbrueckii* subsp. *Bulgaricus* and *Lactobacillus casei*, *Lactobacillus plantarum*, *Lactobacillus acidophilus*, *Lactobacillus delbrueckii* subsp., *Bifidobacterium breve*, *Bifidobacterium longum*, *Bifidobacterium infantis*, and *Streptococcus salivarius* subsp. *Thermophilus.* The selected strains of the genus Lactobacillus, especially when used in combination with other probiotics, can effectively support weight reduction in children and adolescents [117,118]. Other combinations of strains, such as *Bifidobacterium longum*, *Lactobacillus bulgaricus*, and *Streptococcus thermophilus*, also contributed to the improvement in metabolic indices. An association between probiotic use and a reduced BMI was shown with favorable results in inflammatory markers, lipid markers, glucose metabolism, and liver measurements. The variety of species used and their combinations, and the selection of appropriate doses, influenced the results obtained [119,120,121,122,123,124].

A major problem in the context of children’s supplementation is the lack of adequate regulation of active ingredients in dietary supplements. Analyses indicate that there is a risk of contamination of these products with doping substances, which may pose health and legal risks [92]. Therefore, supplementation should only be carried out under the supervision of a specialist, and the products used should come from certified sources [80,98]. The lack of long-term data and regulation means that children who use supplements are exposed to potential health risks, which requires close monitoring and further research [80].

The safety of dietary supplements for physically active children with obesity is an issue of growing concern. Under current food laws, supplements are classified as foods, making them widely available on the market. Their extensive advertising often sparks the interest of consumers, including parents of physically active children, who are looking for ways to improve their children’s fitness and health. In physically active children with obesity, the use of dietary supplements aimed at enhancing athletic performance should be avoided, especially under 15 years of age, according to generally accepted recommendations. It is crucial to ensure a well-balanced diet, which is rich in natural nutrients that promote regeneration and proper development of the body. Children with obesity who undertake regular physical activity can benefit from natural sources of whey protein, maltodextrins, fatty acids, vitamins, and minerals to support proper muscle and nervous system function. It is important to remember that any decision to change the diet should be made with a doctor or dietician to suit the individual needs of the child. Adapting the supply of nutrients to the body’s actual needs is crucial not only for improving physical fitness but also for healthy weight reduction and the prevention of nutritional deficiencies.

## 6. Conclusions

Gym training is a valuable supplement to the treatment process of obesity and serves as an effective tool for improving physical fitness while providing tangible benefits across various dimensions of health. It should be prescribed only after obtaining confirmation from the attending physician that there are no contraindications to this form of physical activity. Additionally, it should always be conducted under the supervision of a specialist who will design the exercises with consideration for safety and proper psychomotor development. We recommend the implementation of multidisciplinary care models and prioritize the validation of these recommendations through robust clinical trials. Collaborative, evidence-based strategies are essential to improving patient outcomes and shaping effective health policies.

## Figures and Tables

**Table 1:** Recommendations, indications, and contraindications for strength training in adolescents with obesity.

Part of Training	Intensity/Time	Recommendations	Contraindications
Warm-up	5–10 min Dynamically, with gradually increasing load	General rehabilitation exercises. Aerobic exercises. Exercises targeting the motor preparation of the trained muscle groups. The proposed form may include endurance training on a cycle ergometer, ensuring offloading of the lower limb joints.	Anaerobic exercises. Endurance training on a treadmill is recommended to be avoided.
Proper training	Duration: 20–30 min (up to 12 years of age), then 45 min Repetitions: 10–12 exercises, with 5–15 repetitions per exercise Rest intervals: Short breaks between exercises Rest duration: A 60 s rest is generally assumed, although there is no conclusive data on this Technique instruction: Taught progressively, adapted to the child’s abilities	Low-load exercises that incorporate proper breathing control, with a gradual increase in load. Exercises engaging both agonist and antagonist muscle groups. Up to 10–12 years of age, bodyweight resistance is recommended, with the possible addition of light resistance (e.g., medicine ball) to enhance training variety and develop all motor skills. Exercises involving various types of throws, dribbling, and kicking. Exercise positions should ensure spinal stabilization, particularly of the lumbar spine, in low positions (lying, sitting), semi-high positions (kneeling, squats, supports), and high positions (standing). From 12 to 14 years of age (up to a maximum of 16 years), isometric exercises are progressively introduced in combination with auxotonic contractions (a combination of isometric and isotonic contractions). A gradual increase in load is recommended using resistance bands, resistance tubes, unloaded barbells, dumbbells, or machine exercises.	Exclusion of jumping, hopping, and running on hard surfaces.
Cool-down	5–10 min training regulating Clarke’s parameters (respiration, heart rate, blood pressure)	Breathing exercises to improve muscle flexibility. Exercises in low positions to promote relaxation of activated muscle groups. Rolling with the use of small equipment such as balls, rollers, and foam rollers is also recommended.	Springing, deepening the movement.
General guidelines	Frequency: 2–3 times per week Minimum training duration: 8–12 weeks	Type of exercise: Individual or group sessions, engaging exercises. Recommendation: Alternating strength training with other physical activities. Health monitoring: Use of health-monitoring devices such as wristbands, accelerometers, and devices capable of tracking heart rate (HR).	Avoiding daily strength training.

Own study based on the literature [68,69,75,76,77,78,79,80].
